# Validation of Persian Version of Mindful Attention Awareness Scale (MAAS) in Iranian Women with Breast Cancer

**DOI:** 10.34172/aim.2022.49

**Published:** 2022-05-01

**Authors:** Roghieh Nooripour, Nikzad Ghanbari, Peyman Hassani-Abharian, Laurel E. Radwin, Simin Hosseinian, Saba Hasanvandi

**Affiliations:** ^1^Department of Counseling, Faculty of Education and Psychology, Alzahra University, Tehran, Iran; ^2^Faculty of Education and Psychology, Shahid Beheshti University (SBU), Tehran, Iran; ^3^Institute for Cognitive Science Studies (IRICSS), Department of Cognitive Rehabilitation, Brain and Cognition Clinic, Tehran, Iran; ^4^Center for Health Care Organizational and Implementation Research (CHOIR), VA Boston Healthcare System, Boston, MA, USA; ^5^Department of Psychology, Faculty of Humanistic Sciences, Khorramabad Branch, Islamic Azad University, Khorram Abad, Iran

**Keywords:** Breast cancer, Iranian women, Mindful Attention Awareness Scale (MAAS), Mindfulness

## Abstract

**Background:**

Breast cancer is now the most significant health issue in women, threatening diverse aspects of human health, including mental health and cognitive function. This research aimed to validate the Persian version of Mindful Attention Awareness Scale (MAAS) in Iranian women with breast cancer.

**Methods:**

We gathered data on 229 women with breast cancer in Tehran through convenience sampling. They completed a demographic questionnaire, the Persian version of MAAS, the General Self-Efficacy Scale, and DASS-21. SPSS-22 analyzed the Pearson correlation between the Persian version of MAAS, general self-efficacy, and DASS-21. Also, LISREL 8.8 was used to analyze the internal structure of the MAAS.

**Results:**

Findings from the confirmatory factor analysis (CFA) showed that the model with one factor fits well with the data (_sb_χ^2^=4.29 (*P*=0.36); SRMR=0.058; CFI=1.0; NFI=0.91; IFI=0.95; RFI=0.97; GFI=0.90; RMSEA=0.069). Significant negative correlations were found between MAAS and DASS-21 scores for anxiety (r=-0.51), depression (r=-0.48) and stress (r=-0.49), indicating an acceptable divergent validity. There was also a positive relationship between MAAS and general self-efficacy (r=0.37; *P*<0.01).

**Conclusion:**

The Persian version of MAAS seems to be a valid scale for evaluating the extent of mindfulness of Iranian women with breast cancer.

## Introduction

 Breast cancer is now the most significant health issue in women, threatening diverse aspects of human health, including mental health and cognitive function. It has been estimated that 2.4 million women died of this disease between 1999 and 2015.^1^ More than 1.5 million women (25% of all women with cancer) worldwide are diagnosed with breast cancer.^2^ This disease has spread equally throughout the world.^3^ In 2019, after lung cancer, breast cancer was the second most common type of cancer in women.^4^

 Cancer is a life-threatening illness that causes over 6.7 million deaths per year.^5^ Some risk factors, including gender, age, estrogen, history of family, genetic defects, and unhealthy lifestyles, may increase the disease incidence.^6^ Several factors contribute to this type of cancer, such as urbanization and unhealthy nutrition habit. So, healthy nutrition and a healthy lifestyle have been identified as preventive factors.^7^

 Emotional and psychological issues can arise in women with breast cancer, such as insomnia, anorexia, suicidal thinking, fear of cancer recurrence, and fear of death. Up to one-third of breast cancer patients may experience mental illness more than one year after the initial surgery.^8^ In the last 12 years, related post-traumatic stress symptoms have been identified one year after surgery. Unwanted side effects of adjuvant cancer therapy may also play a moderating role, despite complications for many years following treatment in a large proportion of women. Psychosocial factors may serve as mental illness mediators.^9^

 Considering the health, psychological, social, and functional problems of women with breast cancer,^10,11^ it is necessary to design and use precise instruments to assess patients’ cognitive, behavioral, and physical symptoms. Some psychometric instruments have been developed to assess, diagnose, and help reduce cognitive and behavioral problems, such as stress, in cancer patients,^12^ for example, mindfulness has been found to reduce stress symptoms and affect beneficial immunological and endocrine changes in cancer patients.^13,14^

 Recently, despite increasing clinical research, there have been very few mindfulness assessments and thus no method of evaluating whether some interventions such as Mindfulness Based Stress Reduction (MBSR) effectively promote progress in the mindfulness level. The initial self-reporting measures of mindfulness that have been developed are listed in chronological order: (1) Freiburg Mindfulness Inventory (FMI),^15^ which had one factor and was developed by meditation practitioners and explicitly designed for meditation experience, which would make it challenging to be applied to individuals who do not practice meditation. FMI is a 30-item tool for evaluating moment-by-moment observation and openness to a negative experience and is designed for experienced meditators. (2) Mindful Attention Awareness Scale (MAAS),^16^ a comprehensive overview is included in this article, which also describes why this has been chosen. (3) Kentucky Inventory of Mindfulness Skills (KIMS),^17^ which includes three different subscales measuring different mindfulness skills, namely, observe, describe, and act with awareness. KIMS is a practical and constructive measure for professionals involved in teaching mindfulness skills to their patients. 4) Cognitive and Affective Mindfulness Scale (CAMS),^18^ although not a mindfulness scale, evaluates attention, awareness, acceptance, and present focus. (5) Toronto Mindfulness Scale (TMS),^19^ a concise one-factor scale, was initially developed to be used in combination with meditation. TMS is a 10-item, single-factor tool measuring conscious mindfulness during an instant meditation exercise. (6) Five Facets Mindfulness Questionnaire (FFMQ).^20^ Some specialists have developed FFMQ based on five additional subjectivity assessment measures, including FMI, MAAS, KIMS, CAMS, and MQ. After carrying out the analysis, it was determined that the mindfulness construct is based on five different constructs. It was concluded that mindfulness is not a one-factor structure but involves factors like “describing/labeling with words.” (7) Southampton Mindfulness Questionnaire (SMQ),^21^ a range intended to measure the awareness of unsettling ideas and pictures. This particular scale was initially designed for psychosis patients. (8) Philadelphia Mindfulness Scale,^22^ a two-dimensional measure that evaluates two elements of mindfulness; present-moment awareness and acceptance that are not necessarily related. Although each tool uses its terms to explain and understand mindfulness, and while there is a wide overlap between theories, based on the varying results of these different studies, more research is required to determine whether the instrument assesses mindful attention awareness accurately.

 In this regard, one of the most widely used clinical instruments is the MAAS.^23^ It is ideal for assessing focus and perception of the momentary experience of real-life a larger community.

 MAAS is a 15-item self-report measure prepared by Brown and Ryan^16^ to evaluate mindfulness. They showed its efficacy in assessing motivation and health that improved MAAS preoperative perception assessment in a limited sample of cancer patients in an MBSR sample was correlated with decreased mood disturbances and stress.

 MAAS is a one-factorial perception of a structure, focusing on variable attention/awareness in the present moment as an integral feature of the mind. The MAAS scale is a quick and easy test that assesses a person’s capacity to be receptive and attentive to a single factor in experiencing the present moment of everyday life.^16^ Using MAAS does not mean that the person has already meditated, and the basic formula has strong psychometric properties. These characteristics make MAAS the most common tool in research studies to assess mindfulness, such as in depression,^24^ stress,^25^ bulimia,^26^ chronic pain,^27^ or cancer.^28^

 Although clinical populations, including women with breast cancer, require careful evaluation, this scale has few psychometric assessments among cancer patients, especially patients with breast cancer. This raises the question of whether MAAS-assessed alertness is valid in clinical populations compared to observations in populations where the instrument is validated. This is essential given the recognized need for validated mindfulness measures and related research. Moreover, to our knowledge, MAAS has not yet been validated for breast cancer in Iranian women, and this is the first attempt to validate it. Therefore, the current study aimed to evaluate the MAAS psychometric assessment for Iranian women with breast cancer.

## Materials and Methods

###  Participants

 The current study is a descriptive-analytical study in Tehran from November 2018 to April 2019. Sampling was done through a convenience method. As some studies had suggested a total of 200–300 for factor analysis to be acceptable,^29^ 229 women with breast cancer were finally selected.

 We focused on the research question to determine which criteria are crucial for participants to meet to help the study collect the most significant results. Inclusion criteria were age 20 to 70 years, breast cancer in stages 1, 2, or 3, the ability to read and write, no history of specific diseases, and willingness to participate in the research activities. Exclusion criteria were incomplete questionnaire response and reluctance to participate in the study, other types of malignancy, and disease duration less than two months.

###  Instruments

 The researchers employed a demographic characteristics checklist to gather data on marital status, educational status, age group, tumor stage, tumor treatment, and time to diagnosis (days).

####  Mindful Attention Awareness Scale 

 This measure consists of a 15-item that uses a 6-point Likert scale from 1 (nearly always) to 6 (nearly never). Total ratings vary from 15 to 90, with higher scores indicating higher rates of mindfulness. Respondents are asked to read every item and report their daily life experiences. In Iran, Cronbach’s alpha was at 0.90 for the non-clinical population.^30^

###  General Self-Efficacy Scale 

 The scale was developed in 1979 by Schwarzer and Jerusalem and revised in 1981 to have ten items, all of which measure general self-efficacy. The score is based on a four-point Likert scale ranging from 1 to 4, with scores of 10 to 20 showing low self-efficacy, between 21 and 30 showing mild self-efficacy, and scores above 30 showing high self-efficacy. Cronbach’s alpha has been reported at 0.82.^31^ In Iran, Cronbach’s alpha was obtained to be 0.81.^32^

####  Depression, Anxiety, and Stress Scale-21 Items (DASS-21)

 In 1995, Lovibond and Lovibond developed a 21-item scale to assess stress, anxiety, and depression. Each question’s score ranged from zero (it does not happen to me at all or never) to 3 (much or often applicable to me). A validity of 0.77 was reported for this scale by Brown et al.^33^ Each subscale contained seven items whose final score could be obtained by summing the items’ scores to vary from 0 to 21 per sub-scale. In Iran, the reliability of DASS-21 was measured at 0.82 using the Cronbach’s alpha method.^34^

###  Procedure

 This research was divided into two sections: instrument’s translation and cultural adaptation strategies, so MAAS was translated into Farsi (Persian) in the first phase using the back-translation technique, and the second was the assessment of its validity. A sample of 53 patients was used to assess temporal consistency. Finally, no Persian word was unclear for the patients, so no adjustments were made to the translated scale in the final Persian edition of MAAS. Moreover, reliability was estimated by test-retest and Guttman split-half coefficient.

###  Statistical Analysis

 Normality was tested by the Kolmogorov-Smirnov test, which indicated that the variable follows a normal distribution (*P* > 0.05). Demographic characteristics and the Pearson correlation between the Persian version of MAAS, self-efficacy, and DASS-21 were analyzed. Confirmatory factor analysis (CFA) was performed to determine the convergent and discriminant validity. Convergent validity refers to how closely the current scale applies to other factors and other measurements of the same construct. Not only does the construct correlate with relevant variables, but it should not correlate with different, unrelated variables. The determination in the last lines has been referred to as discriminative validity.^35,36^ The single-factor structure was used to analyze the internal structure of the MAAS by LISREL 8.8.

## Results

###  Descriptive Statistic

 The participants’ age ranged from 20 to 70 years (Mean = 36.7, SD = 9.24). MAAS’s mean (SD) was calculated at 58.46 (12.72). Cronbach’s alpha for MAASwas0.86 (CI = 0.82–0.89). There was a significant relationship (*P* < 0.001) between MAAS and educational status. Table 1 indicates the correlation between MAAS and socio-demographic and clinical features.

**Table 1:** Correlation between MAAS with the Socio-demographic and Clinical Characteristic

	**N**	**%**	**M**	**SD**	***F***	***P***
Marital status					1.43	0.22
Married	108	47.57	59.73	13.46		
Single/widowed, divorced	119	52.42	57.31	11.94		
Educational status					7.4	**0.001**
Under diploma	53	23.34	56.07	14.33		
Diploma	77	33.92	55.50	11.68		
Above diploma	97	42.73	62.11	11.74		
Age group					1.09	0.33
≤ 45	79	34.80	57.09	12.11		
46–70	92	40.52	59.82	11.81		
≥ 71	56	24.66	58.21	12.42		
Tumor stage					0.43	0.66
I–II	129	56.82	58.15	12.02		
III	98	43.17	58.86	12.37		
Tumor treatment					0.97	0.33
Chemotherapy	126	55.51	57.74	11.96.		
Chemo-radiotherapy	101	44.49	59.35	12..89		
Time to diagnosis(days)					0.42	0.67
0–60	132	58.14	58.75	12.05		
> 60	95	41.85	58.05	12.97		

Bold values indicate the significance at the 5% level.

###  Confirmatory Factor Analysis 

**Figure 1:**
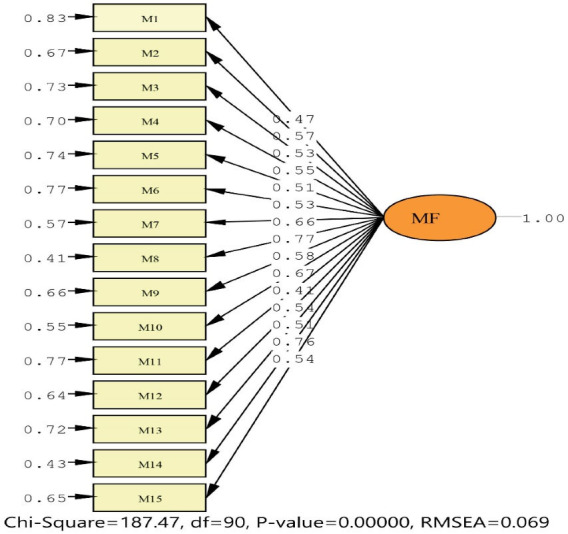
Confirmatory Factor Analysis of Mindful Attention Awareness Scale.

 The results of CFA for MAAS are shown in Figure 1 and Table 2. The CFA findings for a single factor structure are illustrated in Table 3. These findings were reliable in terms of all items.

**Table 2:** Descriptive Statistics for all MAAS Items

**Items **	**Items Statistics**			**Item-Total Statistics**				
	**M**	**SD**	**FL**	**V**	**IT**	**CD**	**SK**	**K**
Item 1	3.77	1.30	0.47	151.29	0.276	0.852	0.339	-0.829
Item 2	4.50	1.42	0.57	141.37	0.543	0.838	-0.584	-0.658
Item 3	3.80	1.44	0.53	143.15	0.480	0.842	-0.039	-0.978
Item 4	3.69	1.53	0.55	140.70	0.518	0.839	-0.016	-1.123
Item 5	3.92	1.56	0.51	145.37	0.371	0.848	-0.168	-1.136
Item 6	4.36	1.54	0.53	143.78	0.423	0.845	-0.455	-1.086
Item 7	4.06	1.54	0.66	137.40	0.612	0.834	-0.273	-1.097
Item 8	4.11	1.49	0.77	135.89	0.680	0.830	-0.237	-1.104
Item 9	3.86	1.55	0.58	140.18	0.523	0.839	-0.234	-1.019
Item 10	3.97	1.53	0.67	137.19	0.618	0.834	-0.212	-1.120
Item 11	2.95	1.41	0.41	153.53	0.179	0.857	0.453	-0.600
Item 12	4.27	1.51	0.54	141.49	0.500	0.841	-0.425	-0.869
Item 13	2.58	1.43	0.51	146.66	0.377	0.847	0.728	-0.086
Item 14	3.91	1.51	0.76	135.85	0.671	0.831	-0.119	-1.136
Item 15	4.64	1.50	0.54	143.50	0.445	0.844	-0.867	-0.385

M, mean; SD, standard deviation; FL, factor loadings; V, scale variance if item deleted; I.T., corrected item-total correlations; C.D., Cronbach’s alpha if item deleted; SK, Skewness; K, Kurtosis.

 The model’s fit indices were evaluated: Root mean square error of approximation (RMSEA; criterion < 0.08) and its 90% confidence, standardized root mean square residual (SRMR; criterion < 0.09), root mean square residual (RMR; criterion < 0.050), comparative fit index (CFI; criterion > 0.90), normed fit index (NFI; criterion > 0.90), incremental fit index (IFI; criterion > 0.90), relative fit index (RFI; criterion > 0.90), adjusted goodness of fit index (AGFI; criterion > 0.80), goodness of fit index (GFI; criterion > 0.90). CFA showed that single factor structure provided a good fit to the data: _sb_χ^2^ = 187.47 (*P*= 0.001); SRMR = 0.058; CFI = 0.95; NFI = 0.91; IFI = 0.95; RFI = 0.90; AGFI = 0.87; GFI = 0.90; RMSEA = 0.069. All items of loads show a significant factor, as shown in Table 3.

**Table 3:** Model Fit Index

_sb_ **X** ^2^	**SRMR**	**CFI**	**NFI**	**IFI**	**RFI**	**AGFI**	**GFI**	**RMSEA**
187.47	0.058	0.95	0.91	0.95	0.90	0.87	0.90	0.069

SRMR, standardized root mean squared residual; CFI, comparative fit index; NFI, normed fit index; IFI, incremental fit index; RFI, relative fit index; GFI, goodness-of-fit index; RMSEA, root mean square error of approximation.

###  Divergent and Convergent Validity 

 There was a significant negative relationship between MAAS and DASS-21 (depression: r = -0.48, < 0.001; stress: r = -.49, *P* < 0.001; anxiety: r = -0.51, *P* < 0.001; and total score: r = 0.52, *P* < 0.001). There was a significant and positive relationship between MAAS and self-efficacy (r = 0.37; *P* < 0.001). These findings illustrate acceptable divergent and convergent validity for MAAS (Table 4).

**Table 4:** Pearson’s Correlation Between MAAS and DASS-21

	**Mean (SD)**	**1**	**2**	**3**	**4**	**5**	**6**
1. MAAS	58.46 (12.72)	1					
2. DASS-21, Depression	17.93 (4.12)	-0.48^**^	0.1				
3. DASS-21, Stress	18.92 (3.72)	-0.49^**^	0.43^**^	1			
4. DASS-21, Anxiety	15.81 (3.85)	-0.51^**^	0.35^**^	0.18^*^	0.1		
5. DASS-21, Total	52.66 (8.59)	-0.53^**^	0.77*^*^	0.69^**^	0.66^**^	1	
6. Self-efficacy	27.32 (6.21)	0.37^**^	-0.41^**^	-0.29^**^	-0.18*	-0.33^**^	1

MAAS, Version of Mindful Attention Awareness Scale; DASS, Depression, Anxiety, and Stress Scale. ^*^ *P* ≤ 0.05; ^**^P ≤ 0.01.

###  Temporal Validity

 A sample of 53 patients were used to assess temporal consistency, and the findings revealed that after two weeks, the coefficient of test and re-test was 0.81 (CI = 0.70–0.75).

## Discussion

 This study demonstrated that the Persian version of MAAS is acceptable in Iranian women with breast cancer to measure mindfulness. There was a significant correlation between the level of education and MAAS throughout our research. Moreover, CFA indicated that higher education level plays a role in increasing women’s awareness of breast cancer among demographic variables. Also, the divergent validity between MAAS with DASS-21 was significantly negative, while the correlation between mindfulness and self-efficacy was positive.

 MAAS has been validated in different clinical populations, such as cancer populations in other countries.^37^ Previous findings have also found that higher scores on this single factor mindfulness measure are correlated with a low mood disorder and student stress.^16^ Growing research has shown that mindfulness could be used widely in many diseases, particularly cancer. The nature of the disease is such that acute stress impairs the patient’s individual and sometimes social functioning and may exacerbate the course of the disease.^38^

 There is a consensus that women have a vast spectrum of physical and mental symptoms over life.^39^ Epidemiologic research has shown several efforts to classify likely symptomatological effects through aging, endocrine modifications, demographics, psychosocial causes, environmental circumstances, cultural disparities, and differences across countries.^40^

 Psychological stress, chronic exhaustion, discomfort, nausea, hair loss, body image disorders, and cognitive decline have been reported by cancer patients.^41^

 Mental and physical conditions and many cancer therapies such as surgery, radiotherapy, chemotherapy, and hormone therapy may affect patients’ family, work, social relationships, and sexual function.^42^ One-third of cancer patients with mental conditions such as anxiety and depression are reported to be dealing with higher percentages of women and youth.^43^ There is also an increasing interest in mindfulness research, especially in oncology situations, to be aware of life stressors such as cancer diagnosis.^44^

 From the cognitive and behavioral point of view, reducing the psychological burden of cancer through cognitive rehabilitation techniques such as mindfulness, in the long run, raises the resilience of the affected women. All variables investigated in this study played an important role in predicting the efficacy of cognitive rehabilitation techniques such as mindfulness. The final model also showed that the higher the education level of the affected women, the more they benefit from the mindfulness technique. Mindfulness can promote different cognitive functions such as retention, thinking, problem-solving, and emotional balance, and these components seem to be better developed in educated women.

 Some studies have shown that mindfulness training could increase awareness and attention in students.^45^ Among clinical populations, such as breast cancer patients, only the educated could increase the performance of this instrument. In other words, present-moment attention and acting with awareness have been more prominent in educated people.

 It is suggested that this study be conducted on different types of cancers and different stages separately to determine the scale’s sensitivity to it. It is also recommended that this study be repeated by changing the population to other chronic diseases. Also, longitudinal research with this scale in women with cancer is recommended to assess their mindful attention awareness.

 While there were strengths in the present study, limitations were also notable. One of the limitations of the present study is that it is limited to women with cancer, limiting generalizability to other statistical populations. Due to the lack of random sampling, generalization of the findings to other populations must be made with caution. So, this scale must be validated in different environments and on different samples in future research. Second, mindfulness is subjective, and it has limitations in its critical measurement. The nature of self-reports fails to eliminate the likelihood of answers influenced by social attractiveness considerations.

 The present study also confirmed that the items used are appropriate, and the questionnaire retains its structure without any possible changes or omissions. Another point is that the cultural and racial differences and different Iranian sample experiences did not cause mindfulness to be evaluated differently from the English language sample. In explaining this issue, we can point to the spiritual context of mindfulness and, consequently, the non-cultural and universal nature of spirituality, free from racial and cultural differences. There is a need for spirituality in all places and times, and spirituality can be defined as a quality beyond gender, nationality, ethnicity, and any other difference.

 This research was done on Iranian women with breast cancer. Future studies can validate the Persian version of MAAS among the general and clinical populations. In practice, this study offers a robust forum for the therapeutic use of MAAS. In potential simulations, though, other latent variables could be possible. Therefore, it is recommended to use other latent variables for future studies. Finally, the validity of Persian MAAS has only started to be evaluated by current research, and future studies could measure the size of useful experimental, neurological, and behavioral findings.

 As a result, the Persian version of MAAS is a helpful tool to measure individual differences in the ability to pay attention and be mindful of momentary experiences among Iranian women with breast cancer; therefore, this scale can be used to evaluate the results of treatments and attention and awareness at the present moment.
